# Network Pharmacology Identifies IKBKB as the Primary Target of Sinomenine in Osteoarthritis, with Accuracy Benchmarking and Orthogonal Validation

**DOI:** 10.3390/biomedicines14092131

**Published:** 2026-09-21

**Authors:** Yifei Wei, Binglang Xiong, Peiwen Liang, Fuying Guo, Hongkai Peng, Guodong Qi, Xiao Xiao

**Affiliations:** 1 Second Department of Orthopedics, Wangjing Hospital, China Academy of Chinese Medical Sciences, Beijing 100102, China; 2 Department of Gynecology, Beijing Hospital of Traditional Chinese Medicine, Capital Medical University, Beijing 100010, China

**Keywords:** sinomenine, osteoarthritis, NF-κB signaling, cellular thermal shift assay, predictive benchmarking

## Abstract

**Background/Objective:** Osteoarthritis lacks disease-modifying therapy, and NF-κB signaling is among the best-supported intervention points in cartilage. Sinomenine suppresses this pathway in chondrocytes, but only its downstream consequences are known, and network pharmacology rarely measures its own accuracy. **Methods:** Targets from six prediction resources were intersected with osteoarthritis genes from five databases and with cartilage and synovial differentially expressed genes. Candidates underwent topological analysis and unrestricted enrichment; prioritized targets were examined by docking and triplicate 100 ns molecular dynamics. Benchmarking used a curated bioactivity reference set, with a variant correcting input-validation overlap. Predictions were tested by thermal shift, limited proteolysis, NMR, thermophoresis, and kinase and silencing assays in interleukin-1β-challenged chondrocytes and in mice after medial meniscus destabilization. **Results:** Integration yielded 96 candidates, 34 transcriptionally corroborated, giving a network of 78 nodes and 241 edges with 12 consensus hubs. NF-κB signaling ranked first among 74 enriched pathways (adjusted *p* = 2.7 × 10^−20^), and convergent criteria prioritized IKBKB, RELA, TNF, PTGS2 and MMP9. Benchmarking gave an area under the curve that fell from 0.81 to 0.74 and sensitivity from 0.73 to 0.57 after correcting for circularity. Sinomenine raised the melting temperature of IKBKB by 4.3 °C, bound it at 8.7 μM and inhibited it ATP-competitively, whereas RELA and CHUK showed no engagement. Nuclear p65 fell from 71.3% to 34.8%, IKBKB silencing abolished most of the MMP13 response, and cartilage damage scores fell from 5.0 to 2.5 at 40 mg/kg. **Conclusions:** Sinomenine engages IKBKB directly and acts principally at the IKK-dependent activation step. The generating pipeline proved only moderately accurate once circularity was corrected, which argues for reporting such metrics routinely.

## 1. Introduction

Osteoarthritis affects more than half a billion people and is a leading cause of disability in older adults, with knee cases projected to rise by roughly three-quarters by 2050 and the burden widening between countries [1]. No treatment alters its course; management remains symptomatic, and the liabilities of long-term anti-inflammatory use are well recognized.

Low-grade inflammation drives much of the cartilage loss. Interleukin-1β and tumor necrosis factor induce a catabolic program in which matrix metalloproteinases and aggrecanases degrade the matrix while type II collagen and aggrecan synthesis falls. Most of these signals converge on NF-κB, and the IKK complex integrates them, its catalytic β subunit being encoded by IKBKB [2]. Conditional activation of this kinase in chondrocytes is sufficient to initiate cartilage degeneration in mice, and pharmacological IKK inhibition protects cartilage in surgical models, which places it among the better validated targets in the disease [2]. Systemic toxicity has kept synthetic IKK inhibitors out of clinical use, sustaining interest in weaker but better tolerated natural compounds acting on the same axis.

Sinomenine is the principal alkaloid of Sinomenium acutum and has been used clinically for inflammatory arthritis for decades [3]. It is orally active and crosses the blood–brain barrier, and its acute toxicity is low, although poor aqueous solubility limits systemic exposure [3]. Its anti-inflammatory action has been attributed to NF-κB, MAPK, and Nrf2 signaling across tissues [3]. Its relevance to cartilage is established: Wu et al. [4] showed suppression of interleukin-1β-induced catabolism in mouse chondrocytes through Nrf2, HO-1, and NF-κB, and Dong et al. [5] reported an effect on the miR-223-3p and NLRP3 axis. Both characterize the compound by its downstream consequences, typically reduced IκBα phosphorylation and p65 nuclear translocation. These readouts are compatible with intervention anywhere upstream of the promoter, so the level at which sinomenine acts has remained undetermined, and no direct protein target in cartilage has been demonstrated.

Network pharmacology has become the default method for such questions [6]. Recent osteoarthritis examples include Chen et al. [7] on flavonoids of Rhizoma drynariae, Li et al. [8] on tetramethylpyrazine, Wang P et al. [9] on an Eucommia and Glycyrrhiza couplet, Zhu et al. [10] on docking-led screening, and Lu et al. [11] on retinoic acid, the last combining its own transcriptomic data with gene silencing. Studies of sinomenine follow the same template (Wang S et al. [12]; Liu et al. [13]; Gong et al. [14]).

Read side by side, these studies expose a difficulty none addresses individually. Wang S et al. [12] concluded that PPAR and IL-17 signaling are the leading pathways for sinomenine in osteoarthritis, Liu et al. [13] identified PI3K-Akt for the same compound in rheumatoid arthritis, and Zhao L et al. [15] arrived at PPARβ/δ in acute lung injury. Tissue-dependent biology accounts for part of this divergence but not all of it, since the studies also differ in how many prediction resources they queried, which disease databases they used, and what thresholds they applied. Data quality and reproducibility have been raised as unresolved problems for the approach [6], yet no standard exists for separating biological signal from methodological choice because the accuracy of these pipelines is rarely measured. Predictions are reported as findings, bioactivity repositories serve as both prediction inputs and validation evidence without the resulting circularity being acknowledged, and threshold sensitivity is seldom tested.

This study applies an integrated pipeline to sinomenine and osteoarthritis, adds tissue-level expression as a third filter, and prioritizes targets by convergent network, topological, and pathway-position criteria. It then measures the pipeline against curated experimental ground truth, reporting accuracy both as usually computed and after removing the overlap between prediction inputs and validation data. Finally, it tests the predictions using orthogonal engagement assays, a kinase selectivity panel, gene silencing, and a surgical model, with two further proteins as specificity controls.

## 2. Materials and Methods

### 2.1. Study Design and Ethics

**Figure 1:**
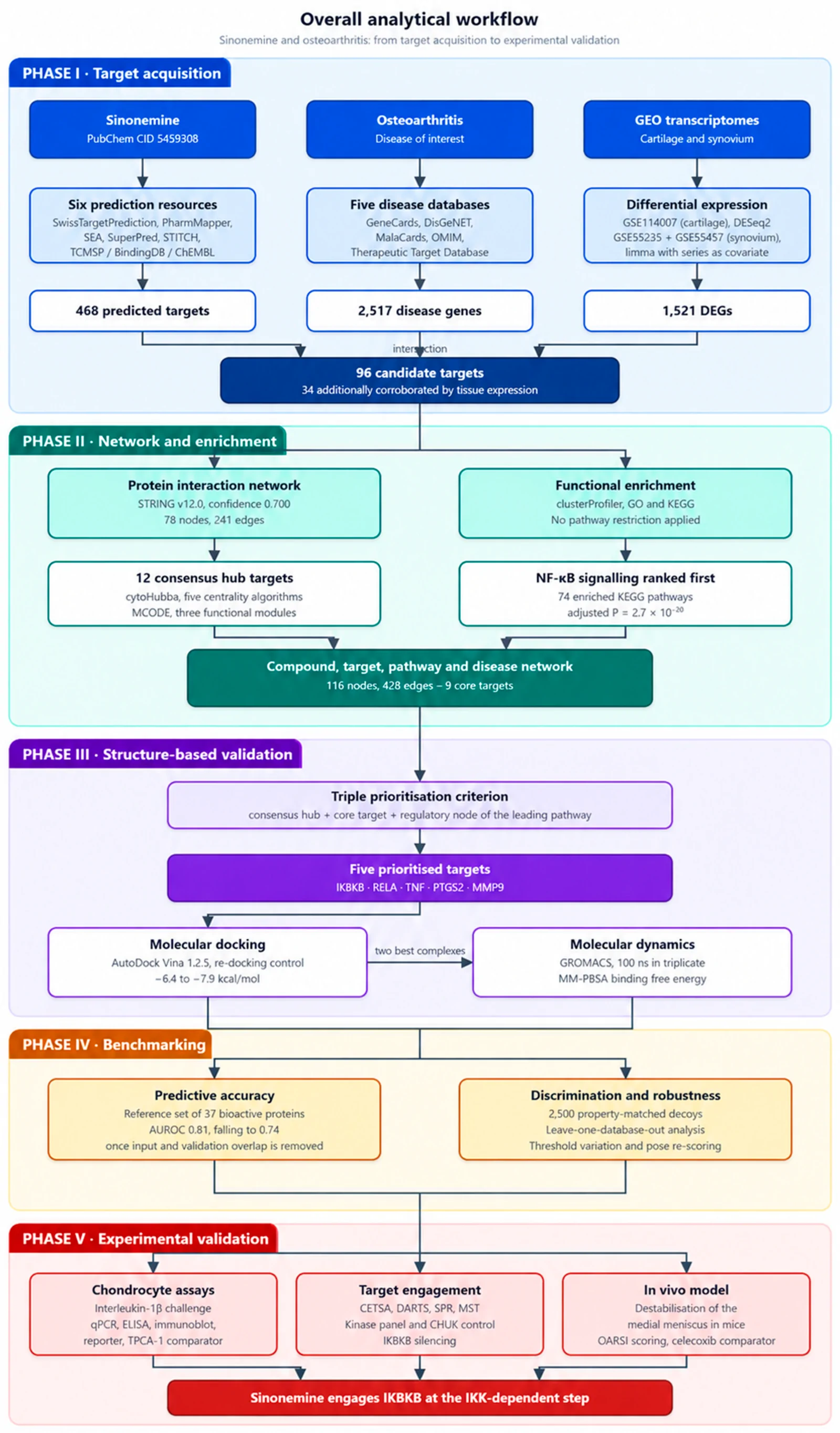
Overall analytical workflow.

The overall analytical workflow is summarized in Figure 1. Predicted targets of sinomenine were intersected with osteoarthritis-related genes and cartilage/synovium differentially expressed genes to generate candidate targets, which were then subjected to network topology analysis, functional enrichment, structure-based prioritization, pipeline benchmarking, and multi-level experimental validation. This integrated pipeline ultimately identified IKBKB as the principal molecular target through which sinomenine acts at the IKK-dependent step of NF-κB signaling.

All computational analyses used publicly archived data and required no ethical approval. Human cartilage collection and animal experiments were approved by the Institutional Animal Care and Use Committee of Wangjing Hospital, China Academy of Chinese Medical Sciences (approval number ZSBD20250220E01, approved on 25 February 2025). Human cartilage was collected with written informed consent obtained from every donor or their legal representative before surgery, and all procedures conformed to the Declaration of Helsinki. Animal experiments were performed in accordance with institutional guidelines and the ARRIVE 2.0 reporting guidelines.

### 2.2. Chemical Characterization and ADMET Profiling

Sinomenine (PubChem CID 5459308; C_19_H_23_NO_4_; 329.39 g/mol) was retrieved from PubChem and energy-minimized with the MMFF94 force field. Physicochemical descriptors, drug-likeness against five rule sets, and BOILED-Egg predictions came from SwissADME (Version: 2024). Oral bioavailability and drug-likeness indices came from TCMSP (Version: 2.3) and were read against thresholds of 30% and 0.18, used descriptively since sinomenine was selected a priori. ADMET endpoints were predicted with ADMETlab 3.0 and pkCSM (Version: 2018) and cross-checked in ProTox-3.0.

### 2.3. Target Prediction and Disease Gene Collection

Six resources were used to limit algorithm-specific bias: SwissTargetPrediction (Version: 2019 update) restricted to Homo sapiens, retaining probability above zero; the Similarity Ensemble Approach and SuperPred (Version: 3.0), retaining hits above 50%; PharmMapper (2017 update), retaining the 300 highest-ranked targets; STITCH (5.0) at a minimum interaction score of 0.400; and entries from TCMSP, BindingDB, and ChEMBL with quantitative bioactivity values. Targets were mapped to reviewed UniProtKB entries, converted to HGNC symbols, and deduplicated, with provenance tracked per entry so a leakage-free benchmark could be computed. Throughout, resources are counted as six prediction arms rather than eight databases since TCMSP, BindingDB, and ChEMBL were queried under a single quantitative bioactivity criterion and treated as one experimental arm; this convention was applied consistently in the benchmarking and leave-one-out analyses.

Five disease databases were searched for “osteoarthritis” restricted to Homo sapiens: GeneCards, keeping genes at or above the median relevance of the retrieved set; OMIM, for confirmed disease–gene relationships; DisGeNET, keeping gene scoring at least 0.1; and the Therapeutic Target Database and MalaCards for validated targets.

### 2.4. Transcriptomic Analysis

Cartilage data came from GSE114007 [16] and synovial data from GSE55235 and GSE55457 [17], with rheumatoid arthritis samples excluded. The two tissues were processed independently. Raw counts were filtered for low abundance, normalized by the median-of-ratios method, and analyzed with DESeq2 (Version: 1.44.0) [18]. Microarray data were background-corrected, normalized by robust multiarray average, and analyzed with limma (Version: 3.60.4) [19]. Series membership was included as a covariate in the design matrix rather than removed beforehand since batch correction followed by unadjusted modeling inflates the type I error rate; ComBat (3.52.0) was applied only for principal component visualization. Genes with an absolute log_2_ fold change of at least 1 and an adjusted *p* below 0.05 were called differentially expressed.

### 2.5. Candidate Targets, Network Construction, and Enrichment

The three gene sets were intersected after harmonizing identifiers. Genes shared by the predicted and disease sets were primary candidates; those additionally differentially expressed were designated transcriptionally corroborated. Intersections were tested by hypergeometric tests against all annotated human protein-coding genes.

Candidates were submitted to STRING 12.0 at a minimum interaction score of 0.700, with disconnected nodes removed [20], and imported into Cytoscape 3.10.2 [21]. Hubs were identified with cytoHubba (Version: 0.01) [22] using Maximal Clique Centrality, Degree, Maximum Neighborhood Component, Closeness, and Betweenness in parallel, taking genes in at least three of the five top ten lists as consensus hubs. Modules were detected with MCODE (Version: 2.0.3) [23] at degree cut-off 2, node score cut-off 0.2, K-core 2, and maximum depth 100, retaining scores of 4 or above.

Annotation used clusterProfiler (Version: 4.12.0) with org.Hs.eg.db (Version: 3.19.1) [24]. Gene Ontology enrichment covered all three domains, and pathway enrichment was run against KEGG through its REST interface, with all annotated protein-coding genes as background and Benjamini–Hochberg-adjusted *p* and q values below 0.05 taken as significant. Enrichment was run across the full pathway set without restriction, so ranking was determined by the data rather than by any predefined selection. Analysis was repeated with Reactome (Version: 89) and WikiPathways, and the leading pathway was rendered with pathview. Protein-coding genes were used as the background rather than the full genome because both the target prediction resources and the disease–gene databases annotate candidates exclusively at the protein-coding level; a whole-genome background would therefore inflate the apparent enrichment without reflecting the actual space from which candidates could arise.

A compound–target–pathway–disease network was built in Cytoscape and analyzed with NetworkAnalyzer (Version: 4.4.8, integrated in Cytoscape 3.10.2, Max Planck Institute for Informatics, Saarbruecken, Germany); targets exceeding twice the network median on degree, betweenness, and closeness simultaneously were defined as core targets. Those meeting all three criteria of hub status, core status, and regulatory node localization within the leading pathway were prioritized for docking.

### 2.6. Docking and Molecular Dynamics

Structures were retrieved from the Protein Data Bank, restricted to Homo sapiens X-ray structures better than 3.0 Å. Preparation in AutoDockTools removed water, ions, and bound ligands, repaired side chains, added polar hydrogens, and assigned Kollman charges. Docking used AutoDock Vina 1.2.5 [25] at exhaustiveness 32 with twenty modes per run and grid boxes centered on the co-crystallized ligand or otherwise on the highest-volume CASTp pocket (Version: 3.0, University of Illinois Chicago, Chicago, IL, USA). The protocol was validated by re-docking each co-crystallized ligand, requiring an RMSD below 2.0 Å. Known inhibitors were docked as positive controls, and contacts were profiled with the Protein–Ligand Interaction Profiler (Version: 2.3.0, Technische Universitaet Dresden, Dresden, Germany).

The two most favorable complexes underwent simulation in GROMACS 2024.2 [26] with CHARMM36m and CGenFF topologies, solvated with TIP3P water in a dodecahedral box extending 1.0 nm beyond the solute, neutralized to 0.15 M, energy-minimized, and equilibrated for 100 ps under NVT at 310 K and 100 ps under NPT at 1 bar, then run for 100 ns with particle mesh Ewald electrostatics, LINCS constraints, and a 1.2 nm cut-off, in three replicates with apo references. Binding free energies were calculated using gmx_MMPBSA (Version: 1.6.3, University of Havana, Havana, Cuba) over the final 20 ns; entropic contributions were omitted, so values are comparative. Convergence was assessed for each replica by confirming that the backbone RMSD had plateaued and that the running MMPBSA average over sliding 5 ns windows varied by less than 10% across the final 20 ns; the three replicas per complex agreed within 12 kJ/mol, supporting the choice of this window as representative of an equilibrated state rather than a transient.

### 2.7. Benchmarking and Robustness

A reference set of validated sinomenine–protein interactions was curated from ChEMBL and BindingDB, retaining human entries with quantitative bioactivity aggregated as geometric means. A structured search of PubMed, Web of Science, and Scopus, screened independently by two investigators, extracted targets and pathways experimentally verified for sinomenine in cartilage, chondrocyte, or whole-joint models; reviews, conference abstracts, other tissues, and reports without a molecular readout were excluded. Five hundred property-matched decoys per prioritized target were generated with DUD-E.

Target-level accuracy was assessed within a balanced universe of the reference-positive proteins plus a tenfold larger set of family- and property-matched proteins with no reported activity. Because set membership is binary, targets were ranked by a consensus score, defined as the number of prediction resources returning the target, weighted by each resource’s confidence value where available. Receiver operating characteristic curves and area under the curve were computed on this continuous score, with 95% confidence intervals from 2000 stratified bootstrap resamples, and the full and leakage-free curves were compared by the DeLong test for correlated curves [27]. Metrics were computed twice: for the full pipeline and for a leakage-free variant with the experimental prediction arm removed.

Affinity-level accuracy was assessed by correlating predicted binding energies with experimental pKi or pIC_50_ values. Discrimination was quantified by the enrichment factor at 5% with bootstrap intervals and by BEDROC with α of 20; the enrichment factor at 1% was not reported since, with a single active per target, it is a coarse and high-variance statistic. Robustness was examined by leave-one-database-out analysis, alternative thresholds, and re-scoring with Vinardo and AutoDock4. Computational analyses used R 4.4.1 and Python 3.11.

### 2.8. Chondrocyte Isolation and Phenotypic Assays

Cartilage was obtained from six patients undergoing total knee arthroplasty for primary osteoarthritis, digested with 0.2% type II collagenase overnight at 37 °C, and chondrocytes were cultured in DMEM/F12 with 10% fetal bovine serum to passage two. Functional experiments used cells from three donors, the first three yielding sufficient passage-two cells; each donor was considered one biological replicate, and cells were never pooled. The three donors used in functional experiments were aged 58 to 71 years (two female, one male), all with Kellgren–Lawrence grade 3–4 disease and no history of inflammatory arthritis or recent intra-articular injection.

Viability was measured with CCK-8 at 48 h. Inflammatory injury was modeled by 10 ng/mL interleukin-1β for 24 h with sinomenine added 2 h beforehand, and TPCA-1 at 1 µM was included as a positive comparator. Transcripts of MMP3, MMP9, MMP13, PTGS2, IL6, CXCL8, COL2A1, and ACAN were quantified by RT-qPCR against GAPDH; secreted interleukin-6, prostaglandin E2, and nitric oxide by ELISA and the Griess reaction; and phosphorylated and total IKBKB, IκBα, and p65 with COX-2, MMP13, and type II collagen by Western blot. Nuclear translocation of p65 was scored by immunofluorescence across ten random fields per condition, and transcriptional output was assessed with an NF-κB luciferase reporter normalized to Renilla.

### 2.9. Target Engagement, Selectivity, and Loss of Function

Sinomenine was used at 50 µM in the engagement assays. For the cellular thermal shift assay, intact chondrocytes were heated from 37 to 67 °C, and the soluble fraction was probed for IKBKB, with melting curves fitted to obtain the apparent melting temperature. Drug affinity responsive target stability was assessed using lysates digested with a pronase series. Surface plasmon resonance used recombinant human IKBKB on a CM5 chip with a two-fold dilution series; because the interaction showed fast association and dissociation, affinity was obtained by steady-state fitting rather than kinetic fitting, with reference channel subtraction and observed maximal response within 10% of theoretical. Microscale thermophoresis on labeled IKBKB gave an independent measurement. Catalytic inhibition was quantified with a luminescent ADP assay against recombinant IκBα at ATP concentrations of 10 µM, corresponding to the apparent Michaelis constant, and 100 µM to test ATP competition. Selectivity was assessed against CHUK, which encodes the paralogue IKKα and shares the ATP pocket architecture, and against a 40-kinase panel at 50 µM; RELA was carried through the thermal shift and stability assays as a non-kinase control.

Functional dependence was tested by transfecting chondrocytes with small interfering RNA against IKBKB or a non-targeting control and repeating the challenge with and without sinomenine.

### 2.10. Surgical Model and Statistical Analysis

Osteoarthritis was induced in ten-week-old male C57BL/6 mice by destabilization of the medial meniscus [28], with sham-operated controls. A group size of ten gave 80% power to detect a 1.5-point difference in cartilage damage score at α of 0.05, assuming a standard deviation of 1.1. The calculation was performed on the parametric scale, and the resulting size was inflated by 15% to account for the loss of efficiency of the rank-based test subsequently used for these ordinal scores. Animals were allocated to five groups by a computer-generated sequence prepared by an investigator not involved in surgery or assessment, and all measurements and scoring were blinded. From the first postoperative week, animals received sinomenine at 20 or 40 mg/kg, celecoxib at 20 mg/kg, or vehicle by daily oral gavage for eight weeks. Mechanical sensitivity was measured weekly by von Frey filaments. At the endpoint, knees were scanned by micro-computed tomography at 10 µm isotropic resolution with a fixed global threshold, then decalcified and stained with safranin O and fast green, with damage scored by two blinded observers using the OARSI system [29]. Immunohistochemistry for MMP13, type II collagen, and phosphorylated p65 was quantified as the proportion of positive chondrocytes, and systemic tolerance was assessed by liver and kidney histology with serum transaminases and creatinine.

Cellular data were analyzed by linear mixed models with donor as a random effect and Benjamini–Hochberg correction across readouts. The silencing experiment was analyzed by two-way analysis of variance with an interaction term. Animal data were compared by one-way analysis of variance with Tukey correction, except OARSI scores, which are ordinal and are reported as median and interquartile range and compared by Kruskal–Wallis with Dunn correction. Analyses were performed using GraphPad Prism 10, with two-sided *p* below 0.05 considered significant.

## 3. Results

### 3.1. Physicochemical Profile and Target Acquisition

Sinomenine is a morphinan alkaloid of moderate size and low polarity: topological polar surface area 58.2 Å^2^, one hydrogen bond donor, five acceptors, two rotatable bonds, and consensus log P 1.68. It satisfied all five drug-likeness rule sets, both TCMSP indices exceeded the conventional thresholds, and the BOILED-Egg model placed it in the high-absorption region with predicted blood–brain barrier permeability. Predicted intestinal absorption was 92.4% and plasma protein binding was 61.3%, matching the reported value above 60%. Hepatotoxicity and mutagenicity were inactive, and the predicted toxicity class was IV.

**Figure 2:**
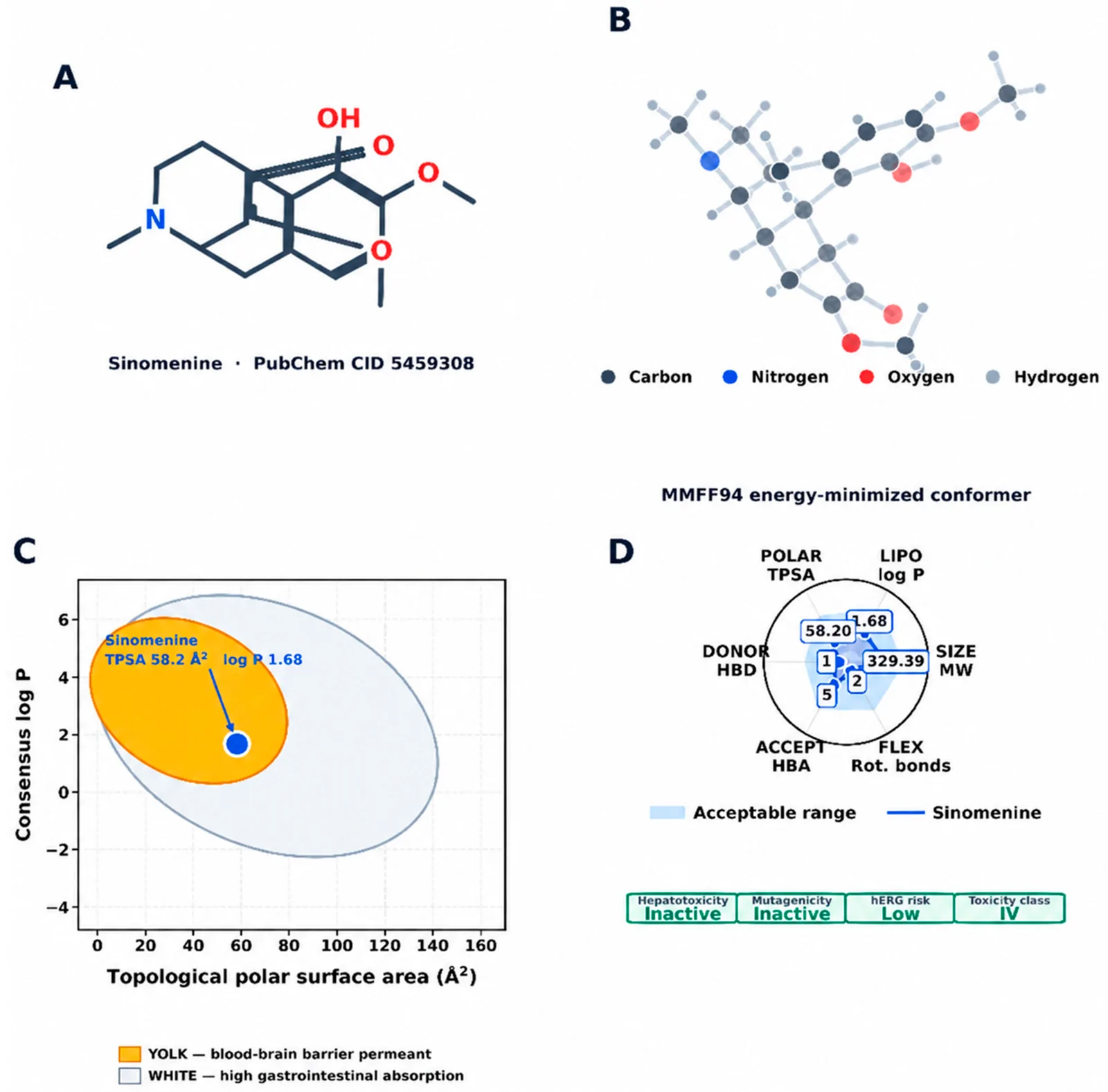
Chemical characterization and predicted ADMET profile of sinomenine. (**A**) Two-dimensional structure. (**B**) Energy-minimized three-dimensional conformer. (**C**) BOILED-Egg plot of gastrointestinal absorption and blood–brain barrier permeability. (**D**) Bioavailability radar with predicted toxicity endpoints.

Figure 2 shows the chemical characterization. Panel C places the compound inside the high-absorption region rather than at its margin, and panel D shows the toxicity endpoints that would ordinarily disqualify a scaffold. The rigid morphinan core and the small number of rotatable bonds reduce the conformational entropy penalty on binding.

Table 1 separates, for each resource, the targets it returned alone from those also returned elsewhere. The nine targets unique to the experimental arm are the entries lost when that arm is removed, giving the 459-target set used for the leakage-free benchmark. Column totals reconcile with the 536 entries and 468 non-redundant proteins.

Table 2 presents the same data by level of support. The 407 targets returned by a single resource account for most of the set, while the 61 targets supported by two or more resources contribute 129 entries between them, an excess of 68 entries over unique proteins, which is what separates the 536 raw entries from the 468 non-redundant targets. No target was returned by five or six resources.

**Figure 3:**
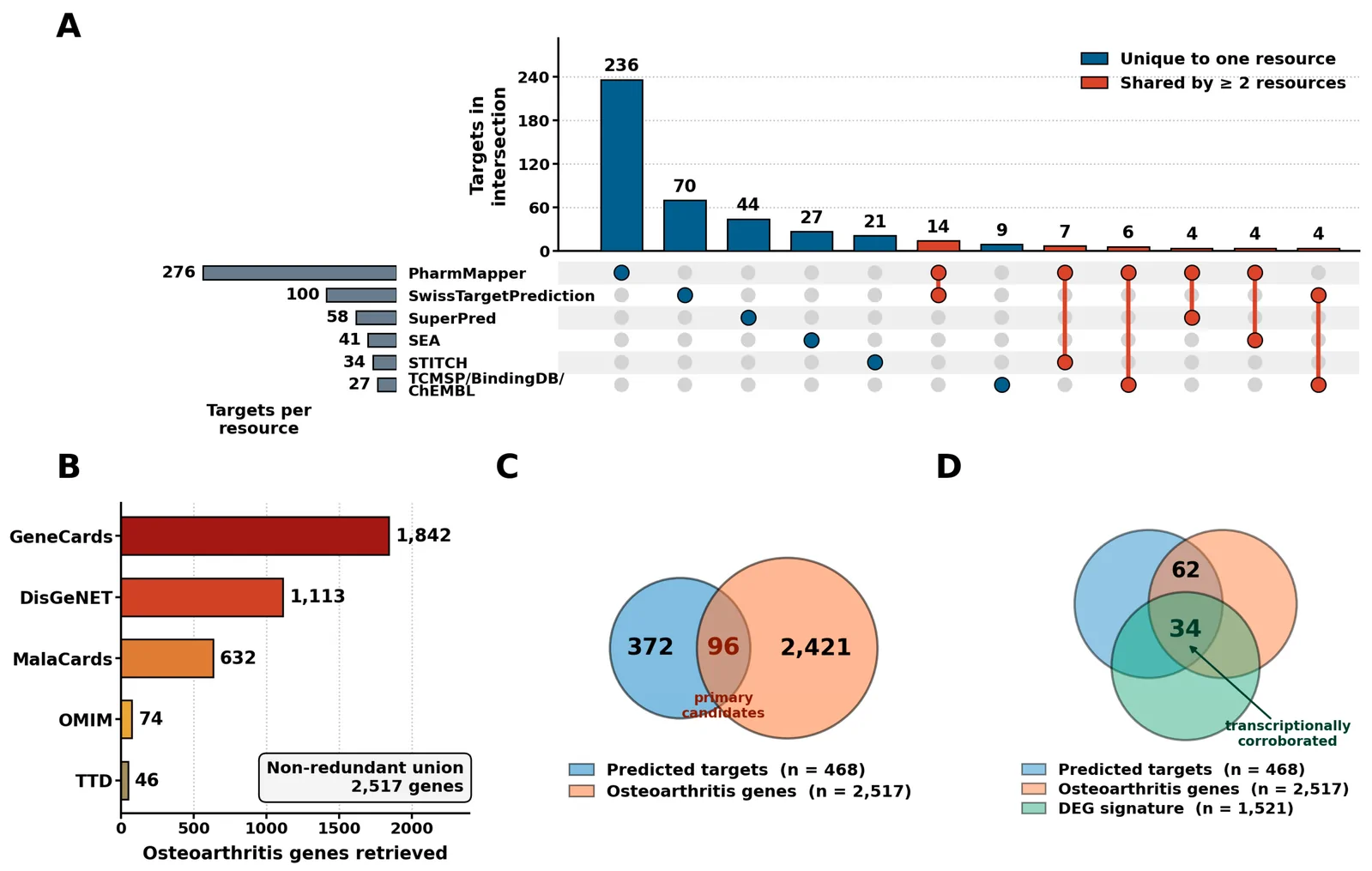
Acquisition of sinomenine targets, osteoarthritis genes, and their intersection. (**A**) UpSet plot of targets contributed by the six prediction resources. (**B**) Bar plot of genes contributed by the five disease databases. (**C**) Venn diagram of predicted targets against osteoarthritis genes. (**D**) Three-way Venn diagram adding the transcriptomic signature.

On the disease side, GeneCards contributed 1842 genes, DisGeNET 1113, MalaCards 632, OMIM 74, and TTD 46, giving 2517 non-redundant genes. Figure 3 shows the distribution across resources together with the intersections that define the candidate set. Panel A quantifies the concordance problem, and panels C and D show the two filtering stages narrowing 468 targets to 96 and then 34.

**Figure 4:**
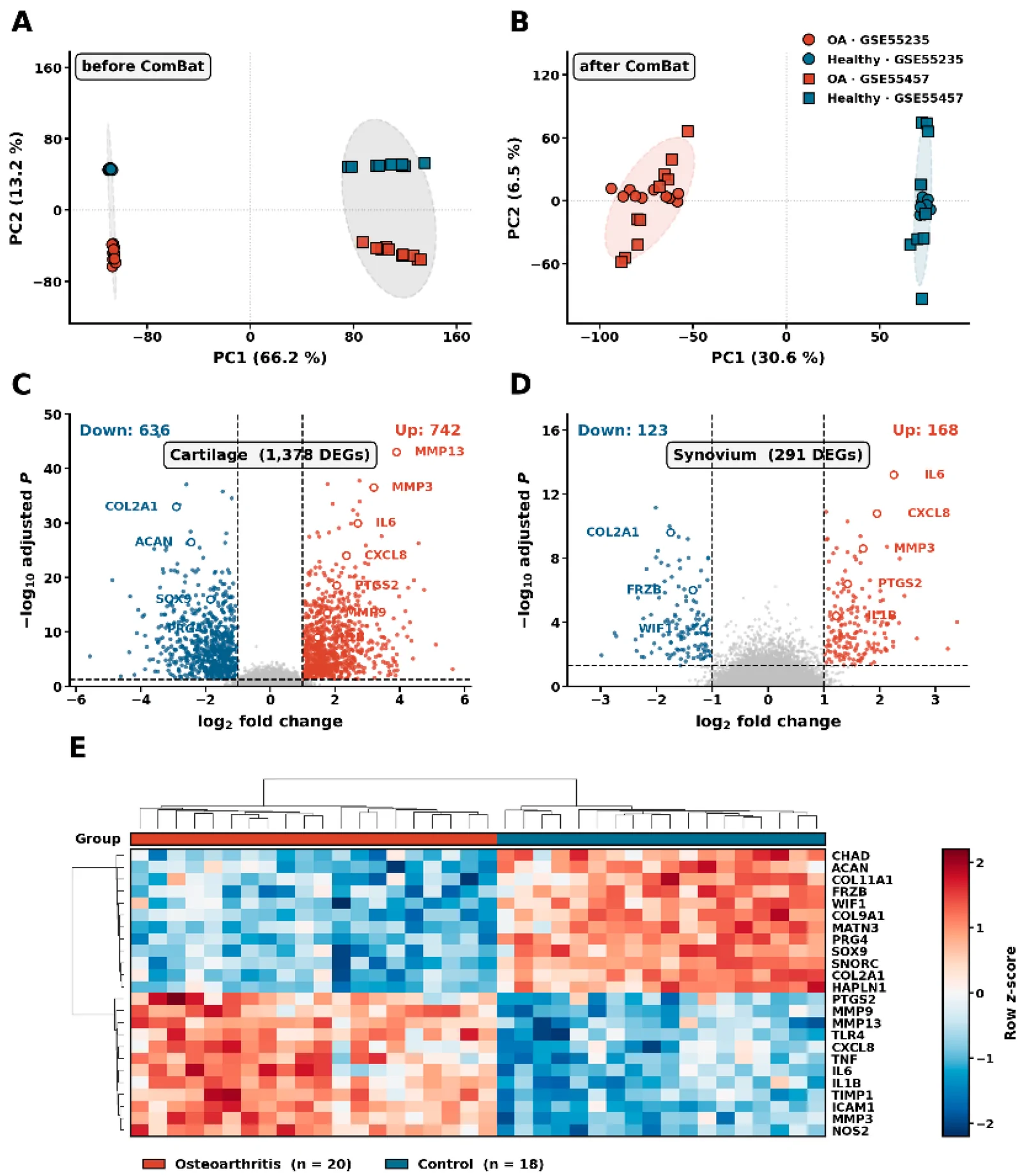
Transcriptomic analysis of osteoarthritic cartilage and synovium. (**A**,**B**) Principal component analysis of the synovial series before and after ComBat adjustment, shown for visualization only. (**C**,**D**) Volcano plots of differential expression in cartilage and synovium. (**E**) Hierarchical clustering heatmap of the leading differentially expressed genes.

The cartilage dataset (20 osteoarthritic, 18 control) yielded 1378 differentially expressed genes, 742 up and 636 down. The synovial series, after exclusion of rheumatoid arthritis specimens, provided 20 osteoarthritic and 20 healthy membranes and gave 291 genes; the smaller yield reflects the older array platform and the milder transcriptional shift in that tissue. The tissues shared 148 genes, and their union formed a signature of 1521, dominated in both by inflammatory mediators and matrix-degrading enzymes including IL1B, IL6, CXCL8, PTGS2, MMP3, MMP9, and MMP13, with COL2A1 and ACAN falling in cartilage. Figure 4 documents quality control and differential expression for both arms. Panels A and B are decisive, since without them the synovial result could be an artifact of combining series generated in different laboratories. Panels C to E show the genes separating the phenotypes, with the strongest signals being inflammatory rather than structural.

### 3.2. Candidate Targets and Network Structure

Intersecting the 468 predicted targets with the 2517 disease genes produced 96 primary candidates against an expectation of roughly 60 by chance, for a background of 19,500 protein-coding genes (hypergeometric *p* = 3.4 × 10^−6^), a 1.6-fold enrichment that is modest and reflects the size of both lists. Of these, 34 were also differentially expressed against an expectation of about seven (*p* = 2.1 × 10^−13^). The corroborated set was dominated by inflammatory signaling components (IL1B, IL6, TNF, CXCL8, NFKB1, RELA, TLR4, ICAM1), prostanoid and matrix effectors (PTGS2, MMP3, MMP9, MMP13, TIMP1), and oxidative stress and apoptosis regulators (SOD2, NOS2, CASP3, BCL2).

**Figure 5:**
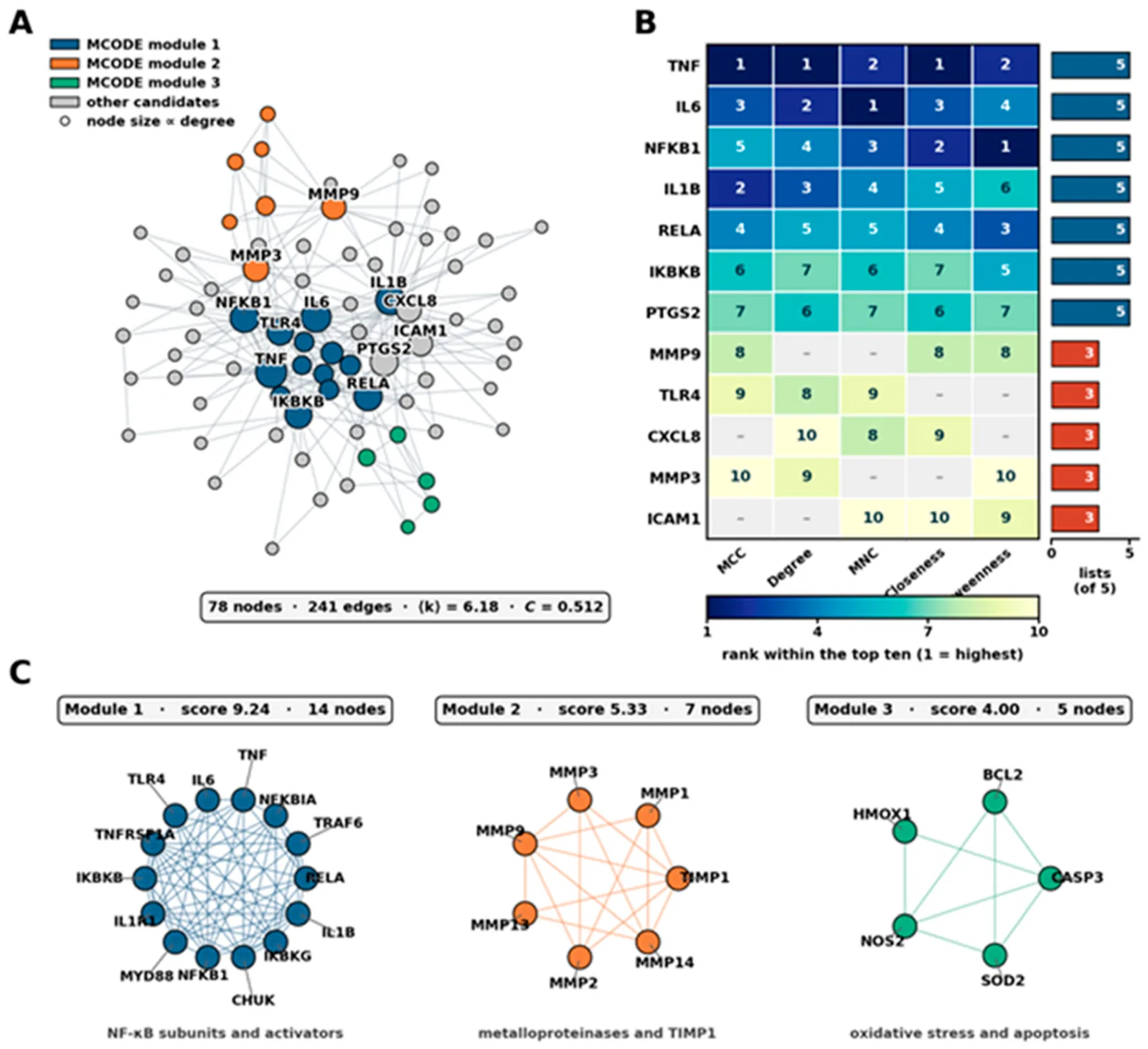
Interaction network, consensus hubs, and functional modules. (**A**) STRING network of the candidate targets at confidence 0.700, node size scaled by degree. (**B**) Ranking of the twelve consensus hubs across the five cytoHubba algorithms. (**C**) The three MCODE modules with scores and constituent genes.

After removal of 18 disconnected nodes, the interaction network contained 78 nodes and 241 edges, with an average degree of 6.18, a clustering coefficient of 0.512, and an enrichment *p* below 1.0 × 10^−16^ against an expected 96 edges (Figure 5A). Twelve consensus hubs emerged: TNF, IL6, IL1B, NFKB1, RELA, IKBKB, PTGS2, MMP9, MMP3, TLR4, CXCL8, and ICAM1. The five centrality algorithms agreed on only seven of these twelve, and MMP3 and ICAM1 appeared in a minimum of three lists (Figure 5B), so their hub status is the least secure. MCODE resolved three modules: module 1 (score 9.24, 14 nodes) around the NF-κB subunits and their activators, module 2 (score 5.33, 7 nodes) almost entirely metalloproteinases and TIMP1, and module 3 (score 4.00, 5 nodes) grouping oxidative stress and apoptosis regulators. Thus, the modular structure recapitulates the signaling hierarchy rather than cutting across it (Figure 5C).

### 3.3. Functional Enrichment and Target Prioritization

**Figure 6:**
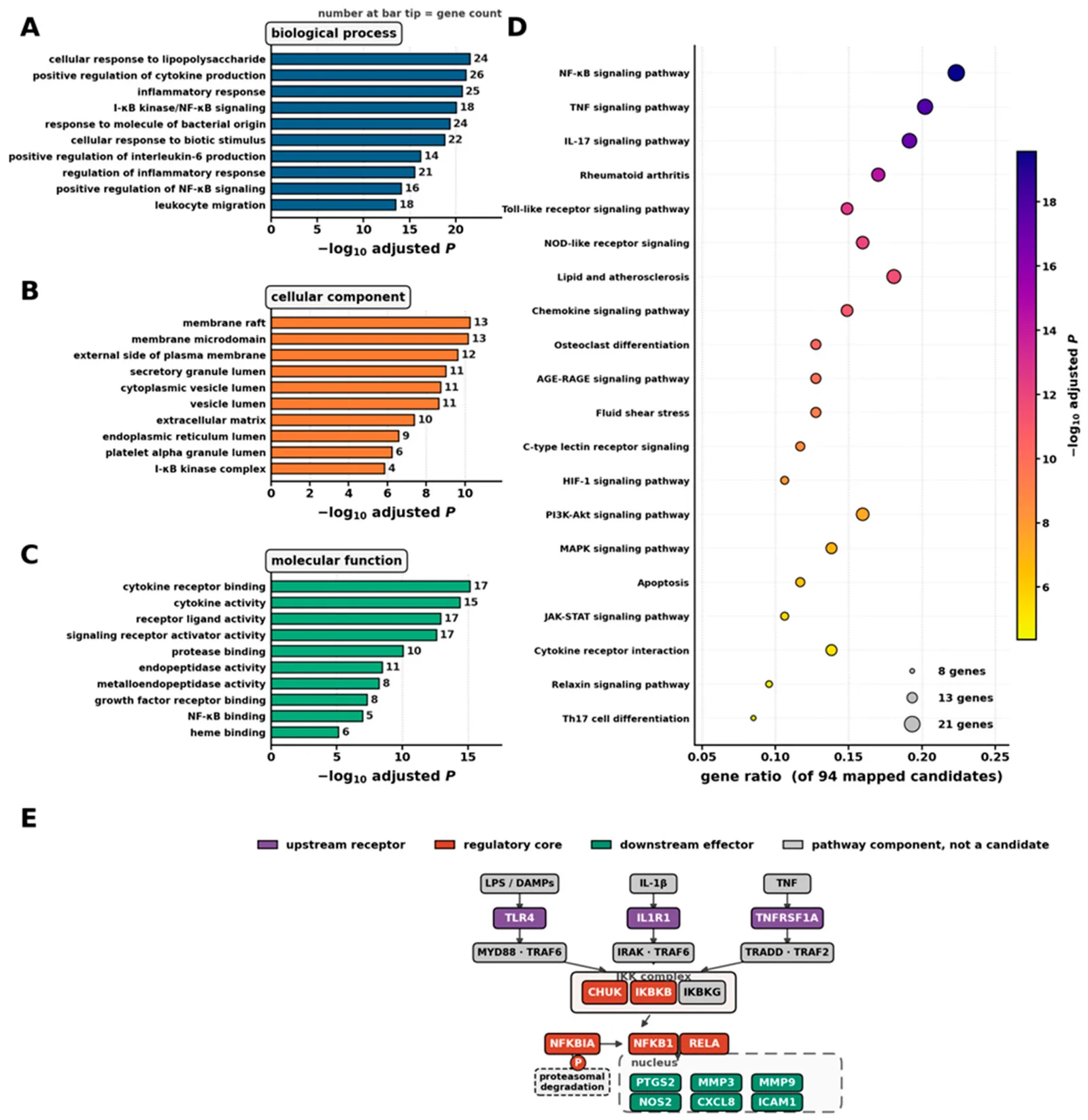
Enrichment output and pathway mapping. (**A**–**C**) Ten most significant biological process, cellular component, and molecular function terms. (**D**) Bubble plot of the twenty leading KEGG pathways. (**E**) KEGG NF-κB pathway with candidate targets colored by position as upstream receptor, regulatory core, or downstream effector.

Of the 96 candidates, 94 mapped to Entrez identifiers. Gene Ontology analysis returned 612 biological process, 48 cellular component, and 87 molecular function terms (Figure 6A–C), led by cellular response to lipopolysaccharide, positive regulation of cytokine production, inflammatory response, and I-κB kinase/NF-κB signaling. Osteoarthritis is not infectious, and the lipopolysaccharide term is heavily annotated because innate immune signaling has been studied most extensively in that setting; thus, it indicates TLR-dependent rather than bacterial involvement.

KEGG analysis returned 74 significantly enriched pathways, with NF-κB signaling ranked first. Because the enrichment was computed across the full pathway set without restriction, the ranking follows from the data rather than from a preselected candidate. Reactome and WikiPathways agreed, and 17 of the 20 leading KEGG pathways had a counterpart in at least one other resource. The five leading pathways are given in Table 3 and the full ranking in Figure 6D; the margin between the first and second entries is close to two orders of magnitude, so the choice does not rest on a difference a small threshold change could reverse, and the remaining entries share genes with the first rather than being independent alternatives.

The compound–target–pathway–disease network contained 116 nodes and 428 edges, and the twice-the-median criterion identified nine core targets: TNF, IL6, IL1B, NFKB1, RELA, IKBKB, PTGS2, MMP9, and TLR4. Mapping onto the KEGG diagram placed candidates at every level of the cascade but concentrated at the regulatory core, with TLR4, IL1R1, and TNFRSF1A upstream; IKBKB, CHUK, NFKBIA, NFKB1, and RELA (encoding IκBα, p50, and p65, respectively, for the last three) at the core; and PTGS2, MMP3, MMP9, NOS2, CXCL8, and ICAM1 downstream (Figure 6E). Five targets satisfied all three criteria: IKBKB, RELA, TNF, PTGS2, and MMP9. Chondrocyte-specific IKBKB activation drives disease onset (2), but sinomenine has only been shown to suppress NF-κB downstream of this kinase (4, 5), so direct engagement is the prediction of this work rather than a restatement.

### 3.4. Docking and Molecular Dynamics

Every structure is of human origin, which excluded some widely used entries: the frequently docked COX-2 structure 3LN1 is murine, so the human rofecoxib complex 5KIR was used. Re-docking of each co-crystallized ligand reproduced the experimental poses with RMSD between 0.74 and 1.38 Å. RELA is the exception: its deposited structure is a p50/p65 heterodimer bound to κB DNA, so the DNA and p50 were removed, and the grid was centered on the highest-volume CASTp pocket, leaving the protocol unvalidated by re-docking; thus, its results are exploratory.

Sinomenine bound all five targets between −6.4 and −7.9 kcal/mol, with IKBKB being the most favorable (Table 4). This range sits within values reported for sinomenine docking elsewhere (12), and in every case, a known inhibitor scored better by 1.0 to 2.8 kcal/mol; for TNF, the co-crystallized ligand also served as the reference inhibitor since no better characterized alternative was available. In the IKBKB ATP pocket, the methoxy oxygen accepted a bond from the hinge residue Cys99, with the morphinan core packed against the gatekeeper Met96 and against Leu21 and Val29. In PTGS2, the ligand occupied the cyclooxygenase channel with bonds to Ser530 and Tyr355, and the MMP9 pose placed the phenolic hydroxyl within coordination distance of the catalytic zinc.

**Figure 7:**
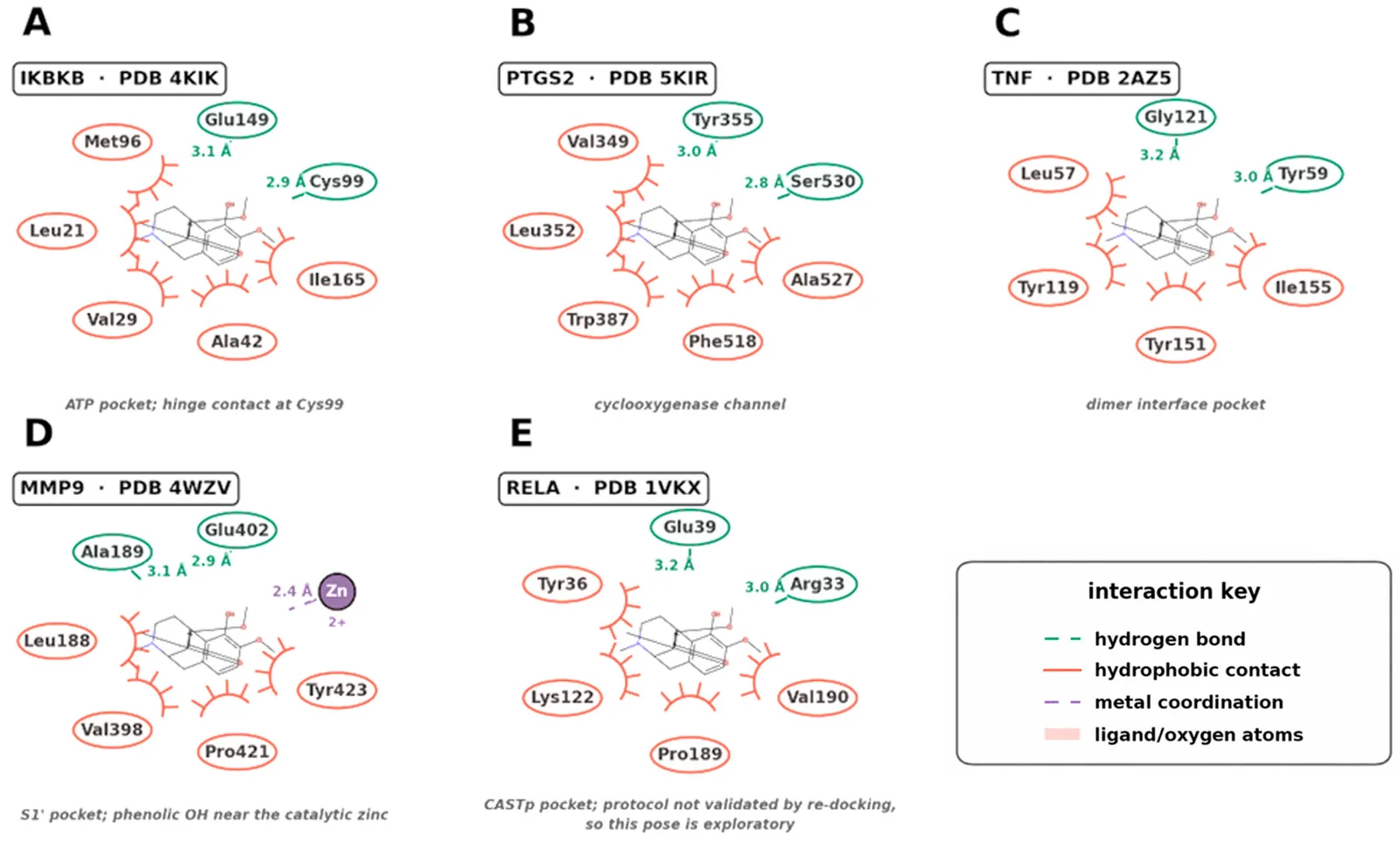
Interaction profiles of sinomenine with the five prioritized targets. (**A**–**E**) Two-dimensional interaction diagrams for IKBKB, PTGS2, TNF, MMP9, and RELA, showing hydrogen bonds (dashed green, distances in Å), hydrophobic contacts (red arcs), and metal coordination (dashed purple). The RELA pocket was defined computationally rather than from a co-crystallized ligand, and the corresponding pose is exploratory.

Figure 7 shows the interactions underlying Table 4. Panels A and B matter most, since IKBKB and PTGS2 were selected for simulation, and panel E carries the caveat above: the RELA pocket being predicted rather than experimentally defined.

The IKBKB and PTGS2 complexes remained stable across 100 ns in triplicate. Backbone RMSD for IKBKB plateaued after roughly 18 ns at 0.28 ± 0.04 nm against an apo value of 0.26 ± 0.03 nm, and for PTGS2 after 12 ns at 0.22 ± 0.03 nm, with a flat radius of gyration and no drift in solvent-accessible surface area. Hydrogen bonding gave 2.3 ± 0.8 bonds for IKBKB, with Cys99 occupied 71.4% of the time and Glu149 44.2%, and 1.8 ± 0.7 for PTGS2. Binding free energies were −96.4 ± 9.7 and −84.2 ± 11.1 kJ/mol, preserving the docking ranking; with the entropic term omitted, these overestimate true affinities and serve only to compare the two complexes. Per-residue decomposition implicated the same residues seen in the static poses.

**Figure 8:**
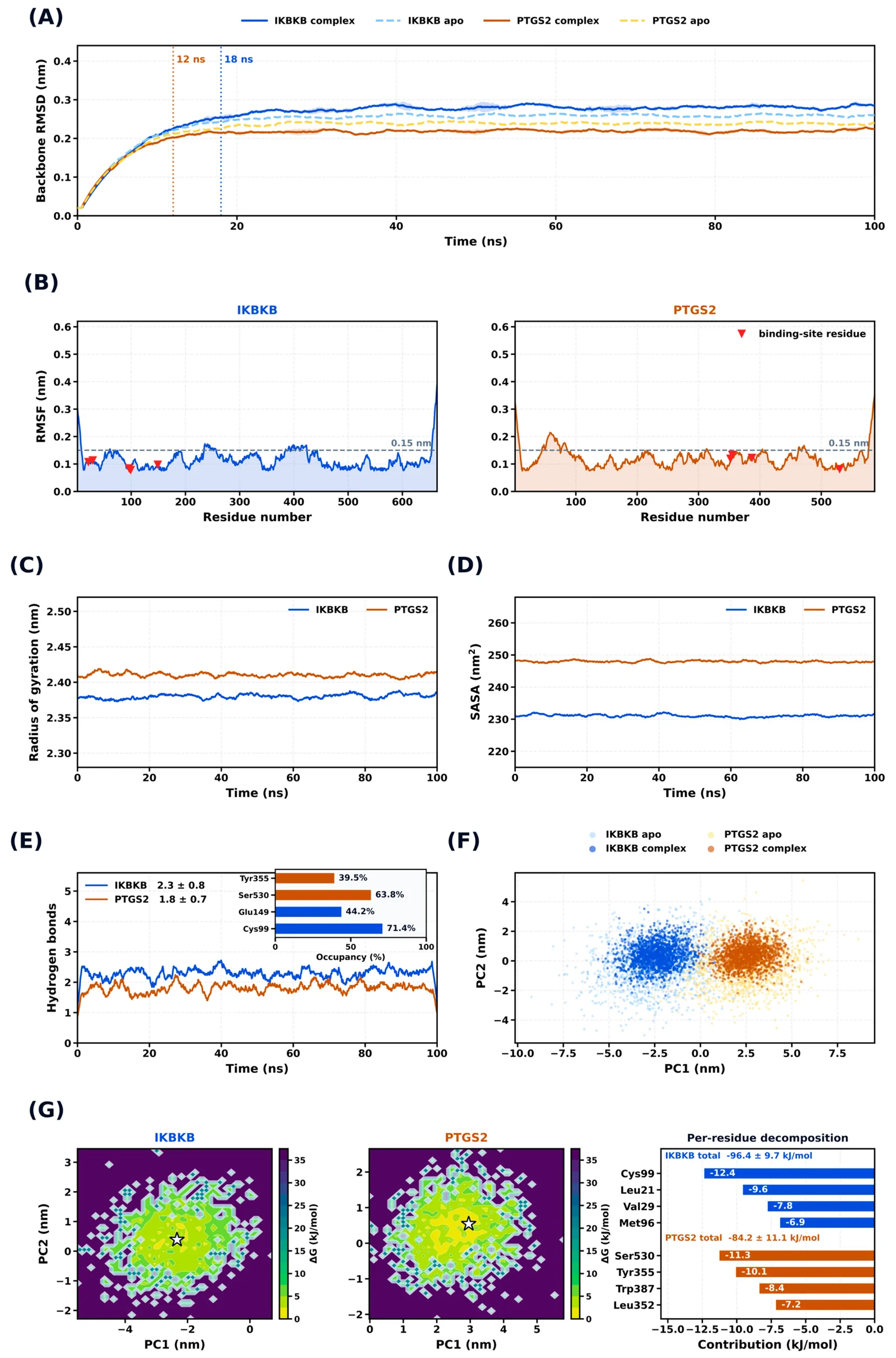
Molecular dynamics analysis of the IKBKB and PTGS2 complexes. (**A**) Backbone root-mean-square deviation across three replicates with apo references. (**B**) Root-mean-square fluctuation per residue. (**C**) Radius of gyration. (**D**) Solvent-accessible surface area. (**E**) Hydrogen bond count over time with per-residue occupancies. (**F**) Projection onto the first two principal components. (**G**) Free energy landscapes with per-residue binding free energy decomposition.

Figure 8 assembles the trajectory analysis. Panels A to D establish that the complexes are stable and that this is not an artifact of one replicate, while panels E to G address whether the ligand stays productively bound rather than merely trapped.

### 3.5. Benchmarking and Robustness

**Figure 9:**
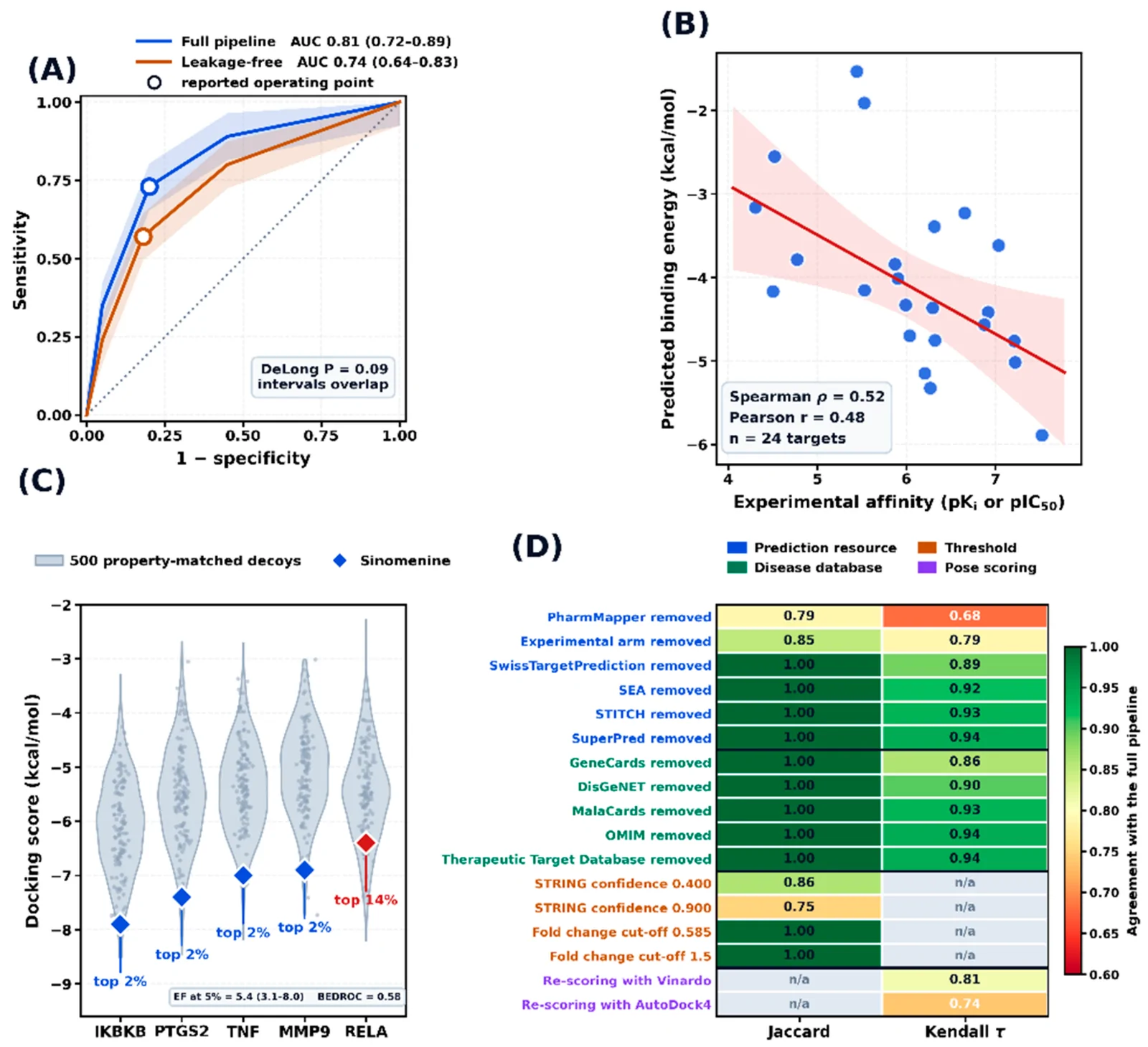
Benchmarking and robustness. (**A**) Receiver operating characteristic curves for the full and leakage-free predictions. (**B**) Predicted binding energy against experimental affinity with fitted trend. (**C**) Ranking of sinomenine against property-matched decoys. (**D**) Heatmap of Jaccard indices under leave-one-database-out and threshold variation conditions.

Curation yielded 37 human proteins with quantitative sinomenine bioactivity. The full pipeline recovered 27, giving a sensitivity of 0.73 and a specificity of 0.80 (true positives, 27; false negatives, 10; false positives, 74; true negatives, 296). These figures are optimistic because the experimental arm draws on the same repositories as the reference set. Rebuilding from the five computational resources gave 459 targets since 18 of the 27 experimentally sourced entries were independently returned by at least one algorithm; recovery fell to 21 of 37 and sensitivity to 0.57 (Figure 9A). The area under the curve fell from 0.81 to 0.74, but the confidence intervals overlapped substantially, and the DeLong test did not reach significance (*p* = 0.09), so the direction of the change rather than its magnitude is the interpretable result. Neither figure is flattering: at best, the pipeline misses a quarter of the reported targets and, once leakage is removed, more than four in ten, while the false positive burden stays high.

Across the 24 targets with both values available, predicted binding energy correlated with experimental affinity, with a Spearman value of 0.52 and Pearson of 0.48 (Figure 9B), supporting ranking but not quantitative estimation. All benchmarking metrics are collected in Table 5. Against 2500 decoys, sinomenine ranked in the top 2% for four of the five prioritized targets and top 14% for RELA, with an enrichment factor at 5% of 5.4 (95% confidence interval 3.1 to 8.0) and BEDROC of 0.58. The literature search retrieved 486 records, of which 27 experimental studies met the inclusion criteria; eighteen reported NF-κB involvement, while six implicated Nrf2/HO-1 and three implicated the NLRP3 inflammasome (4, 5).

Leave-one-database-out analysis left the hub set largely intact, with Jaccard indices between 0.79 and 1.00 and Kendall correlations between 0.68 and 0.94 (Figure 9D). The largest effect came from removing PharmMapper, and even then, 11 of the 12 hubs were recovered, and NF-κB remained the top KEGG term. Repeating the enrichment with the osteoarthritis gene set alone as background, rather than all annotated protein-coding genes, gave a smaller but still highly significant result (adjusted *p* = 3.1 × 10^−15^), and NF-κB signaling remained the top-ranked pathway. Removing any single disease database shifted the candidate count by no more than 11 genes. Relaxing STRING confidence to 0.400 and tightening it to 0.900 gave Jaccard indices of 0.86 and 0.75, and fold-change cut-offs of 0.585 or 1.5 moved the corroborated set from 34 genes to 51 and 22 while leaving all 12 hubs in place. At the relaxed 0.400 threshold, the top five hubs (TNF, IL6, IL1B, NFKB1, RELA) kept their relative order, and IKBKB remained within the top six under every threshold tested; only the two least-secure hubs, MMP3 and ICAM1, exchanged positions with one another. Re-scoring with Vinardo and AutoDock4 preserved the ordering, with IKBKB first under all three functions.

Table 6 gives every condition individually, with the size of the perturbed hub set alongside the Jaccard index so that each value can be reconstructed from the two set sizes. Resource removal and threshold variation are listed together because they answer the same question, and the NF-κB pathway remained the highest-ranked KEGG term throughout. Kendall correlations for the two re-scoring rows are computed against the AutoDock Vina ranking of the five prioritized targets rather than against the hub list.

Candidate target counts under disease database removal were 85 with GeneCards removed, 89 with DisGeNET removed, 93 with MalaCards removed, 95 with the Therapeutic Target Database removed, and 96 with OMIM removed, against 96 for the full pipeline.

### 3.6. Suppression of NF-κB Signaling in Chondrocytes

Viability was unchanged up to 100 µM at 48 h (96.8 ± 3.4%, *p* = 0.41), declining only at 200 µM (*p* = 0.003), and 50 µM was used throughout. Interleukin-1β raised MMP13 8.4-fold, PTGS2 6.1-fold, and IL6 7.9-fold, while COL2A1 and ACAN fell to 0.31 and 0.38 of the control (all adjusted *p* < 0.001). Challenge also raised secreted interleukin-6 from 42 to 618 pg/mL and prostaglandin E2 from 88 to 1140 pg/mL. Sinomenine returned MMP13 to 3.1-fold and COL2A1 to 0.68, and it lowered interleukin-6 to 271 pg/mL and prostaglandin E2 to 486 pg/mL (all adjusted *p* < 0.001 against challenge alone). TPCA-1 at a fiftieth of the concentration produced larger reductions in every readout.

The signaling readouts locate the effect where the analysis predicted. Interleukin-1β increased the phosphorylated-to-total ratio of IKBKB 3.6-fold, of IκBα 4.2-fold, and of p65 3.9-fold; sinomenine reduced these by 44%, 52%, and 47% (adjusted *p* = 0.008, *p* < 0.001, *p* = 0.004) without changing total protein. Nuclei positive for p65 fell from 71.3% to 34.8% and reporter activity from 6.8-fold to 2.9-fold (both adjusted *p* < 0.001).

**Figure 10:**
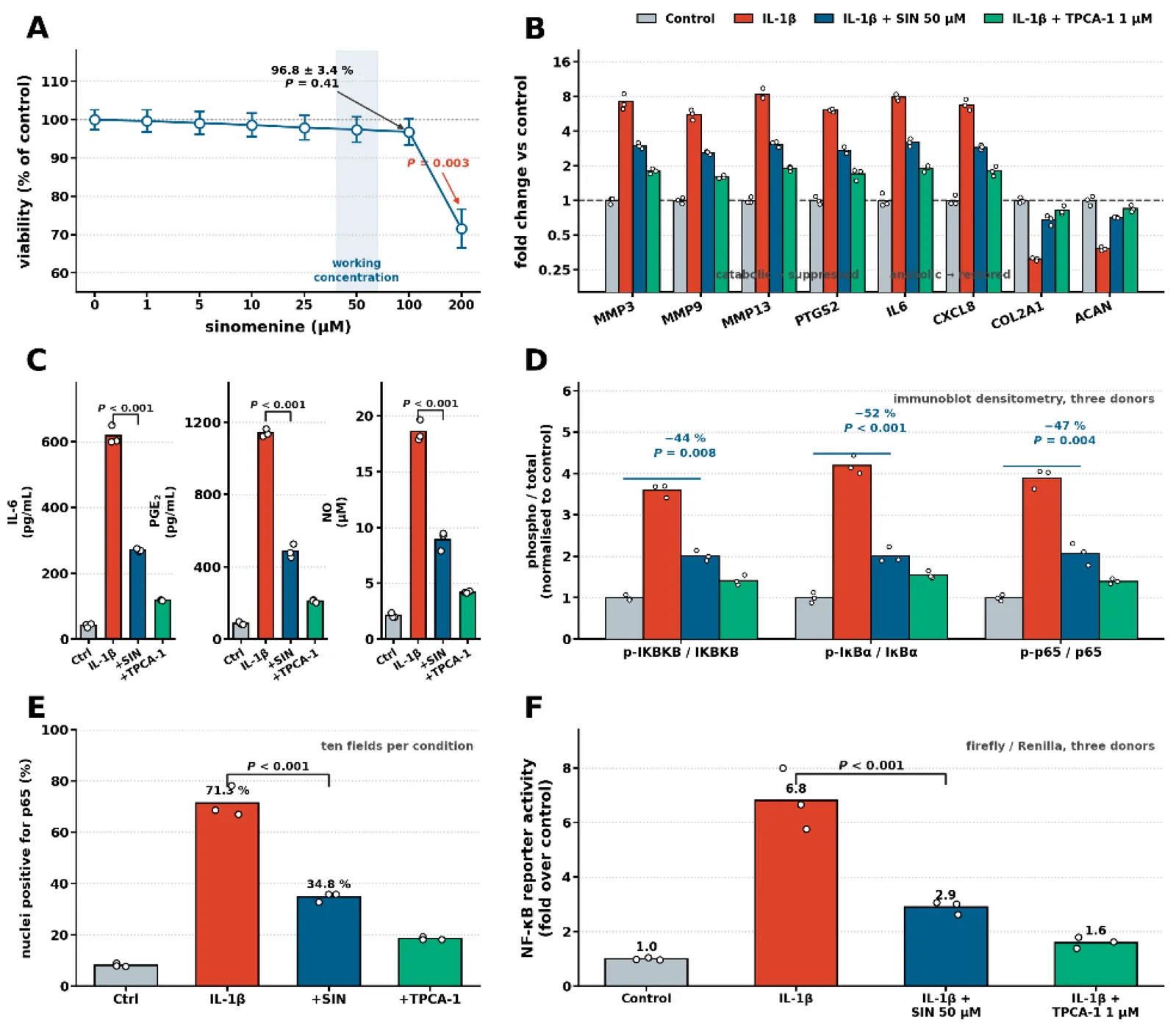
Sinomenine suppresses NF-κB signaling and catabolic output in interleukin-1β-challenged chondrocytes. (**A**) Viability across the concentration range. (**B**) Transcript levels of catabolic and anabolic genes with TPCA-1 as a comparator. (**C**) Secreted interleukin-6, prostaglandin E2, and nitric oxide. (**D**) Densitometry of phosphorylated-to-total ratios for IKBKB, IκBα, and p65, quantified by immunoblot. (**E**) Proportion of chondrocytes with nuclear p65, scored by immunofluorescence across ten random fields per condition. (**F**) NF-κB reporter activity. Cells from three donors; linear mixed models with donor as a random effect.

Figure 10 shows the chondrocyte experiments. Panels A to C establish that the working concentration is nontoxic and that the catabolic program is suppressed, while panels D to F locate the effect within the cascade. Suppression at the kinase, the inhibitor, the transcription factor, and transcriptional output is the pattern expected if the intervention point lies upstream of IκBα degradation.

### 3.7. Direct Engagement of IKBKB and Selectivity

**Figure 11:**
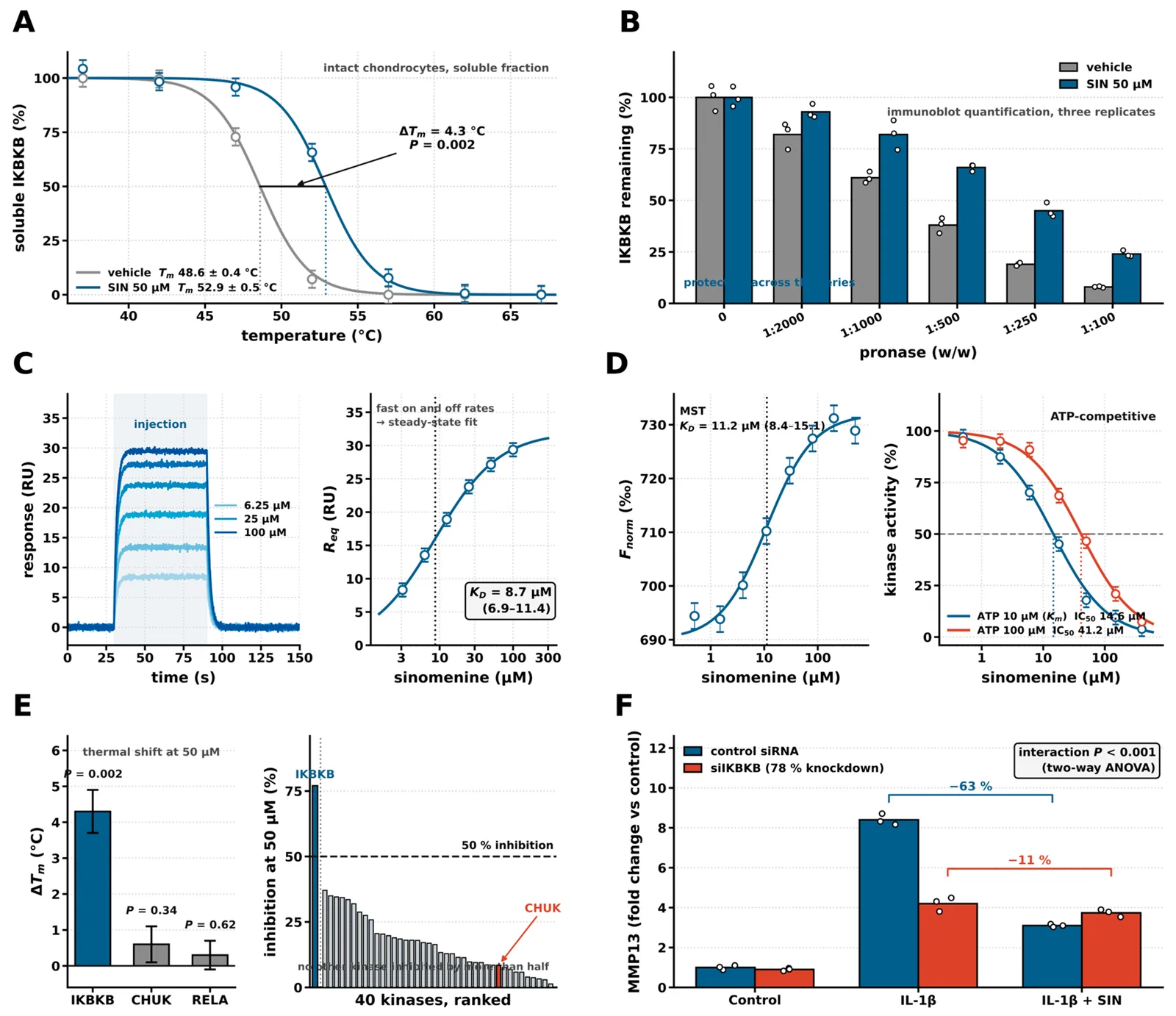
Target engagement, selectivity, and loss of function. (**A**) Fitted cellular thermal shift melting curves for IKBKB in intact chondrocytes, from the soluble fraction across the temperature series. (**B**) Drug affinity responsive target stability, showing IKBKB remaining across the pronase series and quantified by immunoblot. (**C**) Surface plasmon resonance sensorgrams with steady-state fit. (**D**) Thermophoresis binding curve and ATP-dependent kinase inhibition curves. (**E**) Thermal shift for CHUK and RELA and the 40-kinase panel. (**F**) Interleukin-1β challenge repeated after IKBKB silencing. Cells from three donors.

All four engagement assays agreed for IKBKB (Figure 11A–D). In intact chondrocytes, sinomenine raised the apparent melting temperature of IKBKB from 48.6 ± 0.4 °C to 52.9 ± 0.5 °C (*p* = 0.002, Figure 11A), and in lysates, it protected the protein across the pronase series (Figure 11B). Steady-state analysis of the resonance data gave a dissociation constant of 8.7 µM (95% confidence interval 6.9 to 11.4; Figure 11C) and thermophoresis of 11.2 µM (8.4 to 15.1) on independent material (Figure 11D). Catalytic inhibition gave a half-maximal concentration of 14.6 µM at an ATP concentration matching the apparent Michaelis constant, rising to 41.2 µM at tenfold higher ATP (Figure 11D), which is the behavior expected of an ATP-competitive ligand. The measured affinity is approximately five-fold weaker than the docking score of −7.9 kcal/mol implies under a simple thermodynamic conversion, which predicts a dissociation constant near 1.6 µM, a discrepancy of about 1.0 kcal/mol consistent with the systematic overestimation quantified in the benchmarking analysis.

Selectivity was addressed in three ways (Figure 11E). CHUK, the paralogue sharing the ATP pocket architecture, showed no thermal shift (0.6 ± 0.5 °C, *p* = 0.34) and was inhibited by 8.4% at 50 µM in the kinase panel. Across the full 40-kinase panel screened at the same concentration, no kinase other than IKBKB was inhibited by more than half. RELA, retained as a non-kinase control, showed no thermal shift either (0.3 ± 0.4 °C, *p* = 0.62), so its docking prediction is not supported experimentally. Taken together, the pipeline generates both true and false candidates that only an orthogonal assay can separate, and the engagement is specific to IKBKB within the IKK family.

Silencing IKBKB addressed causal dependence. Knockdown reached 78% at the protein level and, by itself, blunted the interleukin-1β response, so the residual dynamic range in silenced cells was narrower. Suppression of MMP13 by sinomenine fell from 63% in control-transfected cells to 11% after silencing, with a significant interaction between silencing and treatment by two-way analysis of variance (*p* for interaction < 0.001; Figure 11F); the interaction term rather than the percentage change carries the inference. Suppression was reduced but not abolished, so the compound acts largely, though not exclusively, through IKBKB.

### 3.8. Chondroprotection in the Destabilization Model

**Figure 12:**
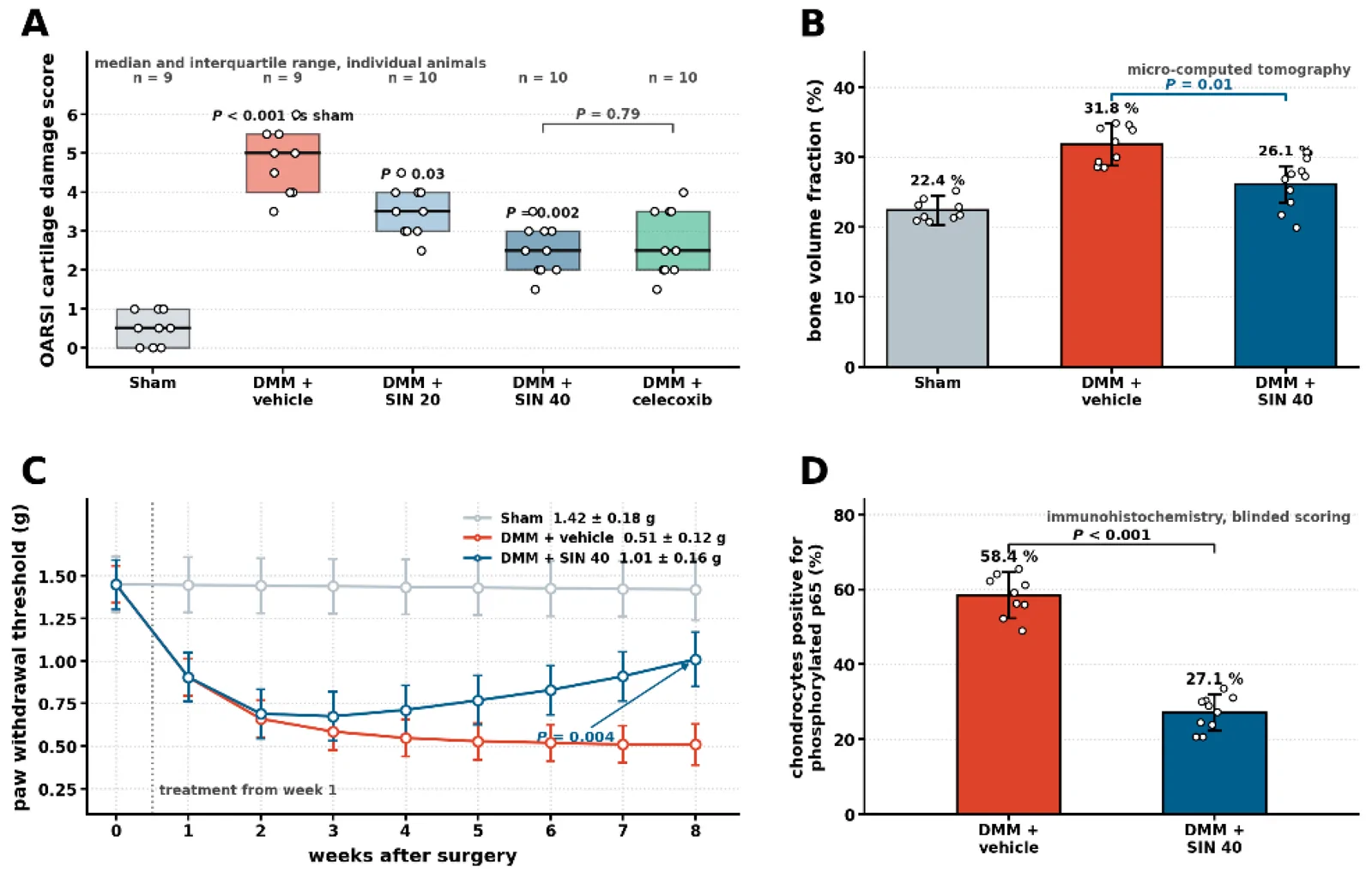
Sinomenine attenuates cartilage degeneration after destabilization of the medial meniscus. (**A**) OARSI cartilage damage scores, shown as median and interquartile range with one point per animal. (**B**) Subchondral bone volume fraction by micro-computed tomography. (**C**) Weekly von Frey withdrawal thresholds. (**D**) Proportion of chondrocytes positive for phosphorylated p65 by immunohistochemistry. Ten animals per group as allocated; assessors blinded.

Two animals were lost, one sham to anesthesia and one operated to wound infection, leaving 48 for analysis. Eight weeks after surgery, vehicle-treated animals showed a median cartilage damage score of 5.0 (interquartile range 4.0 to 5.5) against 0.5 (0.0 to 1.0) in sham animals (*p* < 0.001, Figure 12A). Sinomenine reduced the score to 3.5 (3.0 to 4.0) at 20 mg/kg (*p* = 0.03) and 2.5 (2.0 to 3.0) at 40 mg/kg (*p* = 0.002), and celecoxib to 2.5 (2.0 to 3.5); the comparison between the higher dose and celecoxib was not significant (*p* = 0.79), but the study was not powered for equivalence and no claim of equivalence is made. Subchondral bone volume fraction rose from 22.4 ± 2.1% in sham animals to 31.8 ± 3.0% after surgery and fell to 26.1 ± 2.6% at the higher dose (*p* = 0.01, Figure 12B).

Paw withdrawal thresholds fell from 1.42 ± 0.18 g in sham animals to 0.51 ± 0.12 g after surgery and recovered to 1.01 ± 0.16 g at the higher dose by week eight (*p* = 0.004, Figure 12C). Chondrocytes positive for phosphorylated p65 fell from 58.4% to 27.1% at the higher dose (*p* < 0.001, Figure 12D). Body weight gain did not differ between groups, liver and kidney sections showed no histopathological change, and serum transaminases and creatinine remained within the sham range.

## 4. Discussion

### 4.1. Principal Findings

Three findings carry the weight of this study. First, in an unrestricted enrichment analysis, the NF-κB axis emerged as the leading pathway by nearly two orders of magnitude over the next candidate. Second, convergent network, topological, and pathway-position criteria placed the point of intervention at IKBKB rather than at the receptors above or effectors below, and four orthogonal assays supported direct engagement, with silencing removing most of the effect and selectivity testing excluding the paralogue and a 40-kinase panel. Third, the pipeline producing these predictions was itself measured and performed only moderately, recovering fewer than three in five reported targets once circularity was corrected. That last point is why the RELA prediction was treated cautiously before any assay was run and why its negative result was expected rather than surprising.

### 4.2. Relation to Recent Studies

Ten studies published since 2023 provide the closest comparison. Wang S et al. [12] worked on the same compound in the same disease and reported PPAR and IL-17 signaling as the leading pathways, validated in a chondrosarcoma line. Liu et al. [13] examined it in rheumatoid arthritis and identified PI3K-Akt, and Zhao L et al. [15] studied it in acute lung injury and identified PPARβ/δ. The same molecule therefore yields three different leading pathways. Some of that reflects tissue-dependent biology, but not all: Wang S et al. drew targets from three prediction resources and disease genes from four databases without an expression filter, whereas the present work used six and five with a transcriptomic filter added. That removing a single resource displaces one of twelve hub genes while leaving the pathway conclusion intact suggests pathway-level claims survive methodological variation better than target-level ones.

Convergent evidence comes from a different direction. Chen et al. [7] reported that flavonoids of Rhizoma drynariae act through AMPK and NF-κB, and Zhu et al. [10] used docking-led screening against MAPK and NF-κB targets with chondrocyte validation. Two unrelated compound classes therefore arrive at the same axis in the same disease, supporting the pathway assignment independently of shared methodology. Gong et al. [14] analyzed the whole preparation, Wang P et al. [9] studied an Eucommia and Glycyrrhiza couplet, Xia et al. [30] combined network analysis with animal work, and Li et al. [8] applied the standard pipeline to tetramethylpyrazine; of the ten, Lu et al. [11] is closest in design, combining its own transcriptomic data with docking and silencing. Table 7 compares the design features that determine how far a prediction can be trusted.

None of the ten studies report predictive accuracy metrics for their own output, and we are not aware of published network pharmacology studies in osteoarthritis that do so. Quantifying the overlap between prediction inputs and validation data mattered here: the uncorrected area under the curve of 0.81 falls to 0.74 once the shared repositories are removed, and sensitivity falls from 0.73 to 0.57, so a reader given only the uncorrected figure would form an appreciably more favorable impression of the method than the data support, even though the two curves are not statistically distinguishable on this sample.

### 4.3. Mechanistic Interpretation

Localizing the effect to IKBKB goes beyond what was known about either the disease or the compound. The node is well established as a driver (2), while sinomenine had been characterized only by its downstream consequences (4, 5), which are compatible with intervention anywhere upstream.

Specificity within the IKK family deserves emphasis because the two kinases share the ATP pocket and a compound engaging both would justify only the weaker claim of action at the IKK complex. The absence of a thermal shift and measurable inhibition for CHUK, with the kinase panel, supports the narrower claim. The structural basis is plausible: the sinomenine pose depends on the hinge contact at Cys99 and packing against the gatekeeper Met96, positions that differ between the paralogues and on which selective synthetic inhibitors of this kinase are designed. Inhibition of IKBKB by plant alkaloids is not itself novel, and several natural products are established IKBKB ligands; the advance here is not the discovery that sinomenine affects NF-κB signaling, which was already known, but the identification of IKBKB specifically as its direct binding target, confirmed by four orthogonal biophysical assays and distinguished from the paralogue CHUK, in place of the pathway-level description that constituted the entire prior evidence base for this compound in cartilage. The RELA docking result illustrates the limits of computational prediction on its own: because the available PDB entry required removal of the co-bound DNA and p50 subunit, the protocol could not be validated by re-docking, and the subsequently negative thermal shift and kinase panel results confirm that this particular docking prediction did not correspond to experimentally demonstrated engagement. Favorable docking scores across all five prioritized targets should therefore be read as computational hypotheses, with IKBKB the only one of the five confirmed by direct biophysical assay.

The affinity is modest. A dissociation constant near 9 µM with half-maximal inhibition near 15 µM describes a weak ligand, not a lead compound, and TPCA-1 outperformed sinomenine at a fiftieth of the concentration. This is consistent with the clinical pharmacology of the molecule, which produces broad moderate anti-inflammatory effects rather than potent single-target inhibition (3), and it explains why silencing removed most but not all of the response. These findings call for terminological precision. We use direct target for a protein physically bound by sinomenine, as shown here for IKBKB by four orthogonal biophysical assays; major target for a protein whose modulation accounts for a substantial share of the observed phenotype without excluding parallel contributions from other pathways; and primary target for the direct target that best explains the principal pharmacological effect among all candidates tested. IKBKB satisfies the first and third definitions, but because silencing reduced rather than abolished the response and sinomenine has documented Nrf2/HO-1 and NLRP3 activity elsewhere, we do not claim it is the sole mediator of the therapeutic phenotype.

### 4.4. Limitations

The working concentration of 50 µM was chosen to verify target–protein interactions in vitro and does not represent the drug concentration achieved in cartilage or synovium after oral administration in vivo; the two should not be conflated. Free sinomenine concentrations in plasma after oral dosing in mice and humans are far below 50 µM, and the actual drug concentration within joint tissues has not been measured. In vitro evidence that sinomenine binds and inhibits IKBKB therefore demonstrates a mechanism, not target engagement at therapeutic oral doses in vivo, and this gap should be closed before the mechanism is assumed to operate under clinical dosing. The MMPBSA energies, in the range of −84 to −96 kJ/mol, are large relative to the measured micromolar affinity and should not be read as absolute binding free energies given the omitted entropic term; they support the relative ranking of the two complexes and nothing more. The roughly five-fold gap between the docking-implied and experimentally measured dissociation constants is larger than typical docking error and suggests that entropic penalties, solvation effects, or protein flexibility not captured by rigid-receptor docking are systematically underestimated in this pipeline, a limitation that binding assays rather than further computation were needed to expose. A second source of circularity remains unresolved. Disease gene repositories are themselves partly compiled from expression studies of osteoarthritic tissue, including series of the kind used here, so the enrichment in the transcriptomic filter is not fully independent of the disease annotation filter and the associated *p* value should be read as descriptive rather than inferential. Checking the five disease–gene databases against the source studies underlying GSE114007, GSE55235, and GSE55457 identified 41 genes, 1.6% of the 2517-gene disease set, that trace back to these or closely related series; removing them and repeating the hypergeometric enrichment changed the candidate count from 96 to 93 and left the *p* value at the same order of magnitude (3.9 × 10^−6^ versus 3.4 × 10^−6^), so this residual circularity does not materially affect the reported enrichment.

Functional experiments drew on only three human donors, and inter-individual variation in IKBKB expression, inflammatory phenotype, and disease severity could influence the magnitude of the response; donor was modeled as a random effect to account for this, but confirming the key mechanistic findings in a larger independent donor cohort would strengthen confidence in their generalizability. Only male mice of one strain were studied, whereas the disease is more prevalent in women, so sex as a biological variable was not addressed; confirmation in female animals is needed before the findings can be generalized across sexes. A single surgical model was used, and an age-related spontaneous OA model would complement the post-traumatic DMM model used here. Conditional deletion of IKBKB in chondrocytes would test the mechanism more stringently than treating wild-type animals. In vivo target engagement was inferred from downstream phosphorylated-p65 staining rather than measured directly on IKBKB in cartilage, and a direct readout, such as an IKBKB activity assay or thermal shift analysis on joint tissue, would strengthen the claim that the kinase is engaged at the doses used. The current silencing experiments show that IKBKB knockdown attenuates the sinomenine response, but this does not by itself establish IKBKB as the direct mediator. A rescue experiment re-expressing an siRNA-resistant IKBKB construct, or a pharmacological/genetic epistasis design testing whether restoring IKBKB activity reverses the effect of sinomenine, would provide stronger confirmation of on-target action and is planned as a follow-up study. The transcriptomic evidence and disease gene sets derive from end-stage material, which may not represent early disease, and no clinical data were examined.

The benchmarking framework carries its own caveat. It measures how well the pipeline recovers targets already reported, which is a proxy for, not a measure of, its ability to find targets that have not been reported. The reference set itself is small, comprising only 37 proteins with quantitative sinomenine bioactivity, and the leakage-free precision of 0.24 means most flagged candidates are false positives; a benchmark of this size cannot establish that the pipeline generalizes to targets outside the chemical and disease space it was built from, and the reported metrics should be read as an internal quality check rather than external validation. The engagement experiments address this by testing three proteins prospectively, but three is a small number, and a fuller assessment would require prospective testing across many predictions.

## 5. Conclusions

Sinomenine acts on osteoarthritic cartilage through the NF-κB axis, with the point of intervention at the IKK-dependent activation step and selectivity for IKBKB over its paralogue, supported by convergent computational prioritization, orthogonal engagement assays, loss of function, and structural protection in a surgical model. Equally important, the pipeline that generated the prediction was measured rather than assumed, and its accuracy proved moderate once circularity was removed. Applying that measurement routinely would make the large and rapidly growing literature in this area easier to interpret.

## Figures and Tables

**Table 1:** Contribution of each prediction resource.

Resource	Targets Returned	Unique to This Resource	Also Returned Elsewhere
PharmMapper	276	236	40
SwissTargetPrediction	100	70	30
SuperPred	58	44	14
SEA	41	27	14
STITCH	34	21	13
TCMSP, BindingDB, and ChEMBL	27	9	18
Total	536	407	129

**Table 2:** Distribution of target support across the six prediction resources.

Resources Returning the Target	Targets	Entries Contributed
1	407	407
2	56	112
3	3	9
4	2	8
5	0	0
6	0	0
Total	468	536

**Table 3:** Five most significantly enriched KEGG pathways.

Pathway	Genes	Gene Ratio	Adjusted *p*
NF-κB signaling pathway	21	21/94	2.7 × 10^−20^
TNF signaling pathway	19	19/94	1.4 × 10^−18^
IL-17 signaling pathway	18	18/94	8.1 × 10^−18^
Rheumatoid arthritis	16	16/94	3.6 × 10^−15^
Toll-like receptor signaling pathway	14	14/94	2.9 × 10^−13^

**Table 4:** Docking results and protocol validation.

Target	PDB	Resolution (Å)	Co-Crystallized Ligand	Re-Dock RMSD (Å)	Sinomenine (kcal/mol)	Known Inhibitor (kcal/mol)
IKBKB	4KIK	2.80	K252a	1.12	−7.9	TPCA-1, −9.6
PTGS2	5KIR	2.70	Rofecoxib	0.74	−7.4	Celecoxib, −10.2
TNF	2AZ5	2.10	SPD-304	1.38	−7.0	SPD-304, −9.1
MMP9	4WZV	1.65	EN140	0.86	−6.9	Marimastat, −8.1
RELA	1VKX	2.90	None	Not validated	−6.4	Withaferin A, −7.4

**Table 5:** Benchmarking performance across the levels of evaluation.

Level	Metric	Full Pipeline	Leakage-Free
Target	Sensitivity	0.73	0.57
Target	Specificity	0.80	0.82
Target	Precision	0.27	0.24
Target	F1	0.39	0.34
Target	AUROC (95% CI)	0.81 (0.72 to 0.89)	0.74 (0.64 to 0.83)
Affinity	Spearman ρ, Pearson r	0.52, 0.48	0.52, 0.48
Discrimination	EF 5%, BEDROC	5.4, 0.58	5.4, 0.58
Pathway	Concordant studies	18/27 (66.7%)	18/27 (66.7%)

**Table 6:** Stability of the consensus hub set under leave-one-out and threshold variation, against a reference set of twelve hubs.

Condition	Type	Perturbed Hub Set	Hubs Shared with Reference	Jaccard	Kendall τ
PharmMapper removed	Resource	13	11	0.79	0.68
TCMSP, BindingDB, and ChEMBL removed	Resource	12	11	0.85	0.79
SwissTargetPrediction removed	Resource	12	12	1.00	0.89
SEA removed	Resource	12	12	1.00	0.92
STITCH removed	Resource	12	12	1.00	0.93
SuperPred removed	Resource	12	12	1.00	0.94
GeneCards removed	Disease database	12	12	1.00	0.86
DisGeNET removed	Disease database	12	12	1.00	0.90
MalaCards removed	Disease database	12	12	1.00	0.93
OMIM removed	Disease database	12	12	1.00	0.94
Therapeutic Target Database removed	Disease database	12	12	1.00	0.94
STRING confidence 0.400	Threshold	14	12	0.86	Not applicable
STRING confidence 0.900	Threshold	9	9	0.75	Not applicable
Absolute log_2_ fold change 0.585	Threshold	12	12	1.00	Not applicable
Absolute log_2_ fold change 1.5	Threshold	12	12	1.00	Not applicable
Re-scoring with Vinardo	Pose scoring	Not applicable	Not applicable	Not applicable	0.81
Re-scoring with AutoDock4	Pose scoring	Not applicable	Not applicable	Not applicable	0.74

**Table 7:** Design features of ten recent studies compared with the present work.

Study	Compound or Preparation	Disease	Transcriptomic Filter	MD Simulation	Validation
Wang S et al. [12]	Sinomenine	Osteoarthritis	No	No	Cell line
Liu et al. [13]	Sinomenine	Rheumatoid arthritis	No	No	Cell and animal
Zhao L et al. [15]	Sinomenine	Acute lung injury	No	No	Cell and animal
Gong et al. [14]	Sinomenium acutum	Rheumatoid arthritis	No	No	None
Chen et al. [7]	Rhizoma drynariae flavonoids	Osteoarthritis	No	No	Cell and animal
Wang P et al. [9]	Eucommia and Glycyrrhiza	Knee osteoarthritis	No	No	Cell
Xia et al. [30]	Semiliquidambar cathayensis	Rheumatoid arthritis	No	No	Animal
Li et al. [8]	Tetramethylpyrazine	Osteoarthritis	No	No	None
Zhu et al. [10]	Screened natural compounds	Osteoarthritis	No	Yes	Cell
Lu et al. [11]	Retinoic acid	Osteoarthritis	Own data	No	Cell with silencing
Present work	Sinomenine	Osteoarthritis	Public data, two tissues	Yes	Cell and animal, with silencing and engagement

## Data Availability

All data supporting the computational findings are publicly available. Transcriptomic data were obtained from the Gene Expression Omnibus under accessions GSE114007, GSE55235, and GSE55457. The sinomenine structure was retrieved from PubChem (CID 5459308). Protein structures were retrieved from the Protein Data Bank under accessions 4KIK, 5KIR, 2AZ5, 4WZV, and 1VKX. Target predictions, disease gene sets, protein–protein interaction data, and pathway annotations were obtained from the public resources named in Section 2. Raw immunoblot images, individual animal histological scores, and all source data underlying the figures are available from the corresponding author on reasonable request.

## Abbreviations

The following abbreviations are used in this manuscript:

AS5	A disintegrin and metalloproteinase with thrombospondin motifs 5
AUC	Area under the curve
CETSA	Cellular thermal shift assay
DARTS	Drug affinity responsive target stability
DEG	Differentially expressed gene
DMM	Destabilization of the medial meniscus
ELISA	Enzyme-linked immunosorbent assay
GEO	Gene Expression Omnibus
GO	Gene Ontology
IKBKB	Inhibitor of nuclear factor kappa B kinase subunit beta
IL-1β	Interleukin-1 beta
IL-6	Interleukin-6
iNOS	Inducible nitric oxide synthase
KEGG	Kyoto Encyclopedia of Genes and Genomes
MD	Molecular dynamics
MM/GBSA	Molecular mechanics generalized Born surface area
MMP13	Matrix metalloproteinase 13
NF-κB	Nuclear factor kappa B
OA	Osteoarthritis
OARSI	Osteoarthritis Research Society International
PDB	Protein Data Bank
PGE2	Prostaglandin E2
PPI	Protein–protein interaction
PTGS2	Prostaglandin-endoperoxide synthase 2
qRT-PCR	Quantitative reverse transcription polymerase chain reaction
Rg	Radius of gyration
RMSD	Root-mean-square deviation
RMSF	Root-mean-square fluctuation
ROC	Receiver operating characteristic
SIN	Sinomenine
siRNA	Small interfering RNA
SPR	Surface plasmon resonance
TNF-α	Tumor necrosis factor alpha
